# Metal Oxide Sensors for Electronic Noses and Their Application to Food Analysis

**DOI:** 10.3390/s100403882

**Published:** 2010-04-15

**Authors:** Amalia Berna

**Affiliations:** CSIRO Entomology and CSIRO Food Futures Flagship, GPO Box 1700, Canberra ACT 2601, Australia; E-Mail: amalia.berna@csiro.au; Tel.: +61-2-6246-4258; Fax: +61-2-6246-4000

**Keywords:** food, metal oxide semi-conductor sensor, electronic nose, E-nose

## Abstract

Electronic noses (E-noses) use various types of electronic gas sensors that have partial specificity. This review focuses on commercial and experimental E-noses that use metal oxide semi-conductors. The review covers quality control applications to food and beverages, including determination of freshness and identification of contaminants or adulteration. Applications of E-noses to a wide range of foods and beverages are considered, including: meat, fish, grains, alcoholic drinks, non-alcoholic drinks, fruits, milk and dairy products, olive oils, nuts, fresh vegetables and eggs.

## 1. Electronic Nose and Metal Oxide Semi-Conductor Sensors

The two main components of an electronic nose (E-nose) are the sensing system and the automated pattern recognition system. The sensing system can be an array of several different sensing elements or a single device or a combination of both. Volatile organic compounds (VOCs) presented to the sensor array produces a signature or pattern which is characteristic of the vapor. By presenting many different chemicals to the sensor array, a database of signatures can be build up. Data analysis and pattern recognition (PARC) in particular, are also fundamental parts of any sensor array system. There are a variety of PARC methods available which can be categorized in three classes. The choice of the method depends on available data and the type of result that is required. Graphical analysis with bar charts, profiles polar and offset polar plots are simple forms of data treatment that may be used with an electronic nose. A second way of analysing E-nose signals is by means of multivariate analysis. Multivariate analysis generally involves data reduction. It reduces high dimensionality in a multivariate problem where variables are partly correlated, allowing the information to be displayed in a smaller dimension. There are many multivariate techniques to choose from: principal component analysis (PCA), cluster analysis (CLA), linear discriminant analysis (LDA), partial least squares (PLS), *etc.* A third category is based on artificial neural networks (ANN). A neural network consists of a set of interconnected processing algorithms functioning in parallel. On a very simplified and abstract level, ANN is based on the cognitive process of the human brain [1,2].

Gas sensors, based on the chemical sensitivity of metal oxide semi-conductors (MOS), are readily available commercially. They have been more widely used to make arrays for odor measurement than any other class of gas sensors [1]. Although the oxides of many metals show gas sensitivity under suitable conditions, the most widely used material is tin dioxide (SnO_2_) doped with a small amount of a catalytic metal such as palladium or platinum. By changing the choice of catalyst and operating conditions, tin dioxide resistive sensors have been developed for a range of applications. Materials with improved performance with respect to relative humidity variations have been found by empirical experimentation [3]. Titanium-substituted chromium oxide (CTO) is an example of such a material. Other available oxide-based gas sensors include zinc oxide (ZnO), titanium dioxide (TiO_2_) and tungsten oxide (WO_3_).

In addition to variations in the composition of MOS sensor materials, the metal oxide film deposition is an important variable governing sensor performance design [4]. Deposition techniques include physical or chemical vapour deposition, evaporation and spraying for thin films (6–1,000 nm), a screen printing and painting for thick films (10–300 μm). While thin film devices offer a faster response and significantly higher sensitivities, these are much more difficult to reproducibly manufacture which means that commercially available MOS sensors are often based on thick film technologies [2].

The sensor usually comprises a ceramic support tube containing a platinum heater coil onto which sintered SnO_2_ is coated onto the outside of the tube with any catalytic additives. Gas samples are sensed by the change in the electrical resistance of the metal oxide semi-conductor. Resistance changes due to combustion reactions occurring within the lattice oxygen species on the surface of metal oxide particles [1]. The sign of response (either an increase or decrease in resistance) leads to a simple classification: gas can be classified as either oxidizing or reducing which means that the oxide can be classified as *p* or *n* type. The *n* and *p* type designations indicate which charge carrier acts as the material's majority carrier. *N* types are semi-conducting materials, which are doped with atoms capable of providing extra conduction electrons to the host material. This creates an excess of negative (*n*-type) electron charge carriers. *P* type semi-conductor (*p* for positive) is obtained by carrying out a process of doping, that is adding a certain type of atoms to the semi-conductor in order to increase the number of free charge carriers (in this case positive). For *p* type oxides, an increase in the resistance is found in the presence of reducing gases, while the resistance decreases in response to oxidizing gases; *n*-type oxides show opposite behavior [3]. Examples of *n* type oxides are SnO_2_ and WO_3_; and a *p*-type oxide is CTO.

An electronic nose can detect and estimate odors quickly though it has little or no resemblance to animal noses. Sensors used in E-noses are less independent and are more narrowly tuned to certain VOCs, compared with olfactory receptors from invertebrate animals like fruit flies [5]. Although MOS sensors respond to a broad range of volatiles, they have higher affinity for aldehydes, alcohols and ketones and they are less responsive to molecules like terpenes, aromatic compounds or organic acids. To solve the lack of selectivity, some approaches have been undertaken. One option is to increase the sensitivity and selectivity of the sensor by exploitation of the operating temperatures for the sensor. Thermal cycling of semi-conductor gas sensors take the advantage of the fact that different classes of reducing gases have different reaction rates. For example, carbon monoxide and hydrogen sulfide will oxide on the sensor at relatively low temperatures (starting near room temperature), alcohols and ketones at intermediate temperatures (200 °C and above) and alkanes (propane and methane in particular) at high temperatures (above about 400 °C). Thus sensors run at different temperatures will show a degree of selectivity to each gas without increasing the number of sensors [6]. From this point of view, it has been already shown that, for some applications, a significant improvement in terms of selectivity can be obtained by selecting an appropriate temperature profile, even though this approach has not been exploited for food analysis [7–11]. Another novel option to improve selectivity and independence of sensors is the use of tin dioxide and chromium titanium oxide thick film overlaid with zeolites [12–15]. Zeolites, microporous and aluminosilicate minerals, are ideal for modifying the composition of the gas phase within a porous body. They catalyse and they are size- and shape- specific cracking with partial oxidation.

This review article focuses on the use of MOS-based electronic noses for food applications, the technical limitations for some applications and the different approaches undertaken to overcome them. Problems that have been tried to solve with MOS-based electronic noses are those related to quality control, monitoring process, aging, geographical origin, adulteration, contamination and spoilage (Table 1).

## 2. Application of MOS to Food

### 2.1. Meat

Meat is an ideal growth medium for several groups of pathogenic bacteria. Estimation of meat safety and quality is usually based on microbial cultures. Bacterial strain identification requires a number of different growth conditions and biochemical tests with overnight or large incubation periods and skilled personnel, which means that testing may not be frequently performed. Other methods of determining meat safety involve quantifying volatile compounds associated with the growth of microorganisms on meat but these are also time consuming [16–18]. Winquist *et al.* [19] evaluated pork and beef freshly ground using ten metal oxide semi-conductor field-effect transistor sensors (MOSFETs) with thin catalytic active metals like Pt, Ir and Pd, and four SnO_2_ based Taguchi type sensors (Figaro Engineering Inc, Japan). Compared to MOS sensors, MOSFETs rely on a change of electrostatic potential and they are based on the modulation of charge concentration by a MOS capacitance between a body electrode and a gate electrode located above the body and insulated from all other device regions by a gate dielectric layer which in the case of a MOSFET is an oxide, such as silicon dioxide [1]. A carbon dioxide detector based on infrared ray absorption (Rieken Keiki Co, Japan) was also included in the array. The array of sensors was able to determine the type of meat and predict the storage time as well. When the carbon dioxide monitor was omitted the performance in predicting storage time decreased. Carbon dioxide concentration is an important parameter to consider for predicting shelf life storage of meat products. Balasubramanian *et al.* [20] evaluated the changes in the headspace of vacuum packaged beef strip loins inoculated with *Salmonella typhimurium* using a metal oxide based electronic nose. The E-nose housed seven thick film SnO_2_ MOS sensors (Figaro Engineering Inc, Japan). Microbiological measures as a standard method to determine spoilage level included *Salmonella* counts and total aerobic counts. The average prediction accuracy of the E-nose responses was 69.4% using step-wise linear regression principal components (PC) as input. The accuracy of predicting was improved to 83% when using independent components (IC). PCA is a second-order linear transformation method of data representation which assumes that data follows a Gaussian distribution and uses the variance within the data set to estimate the transform. IC analysis is a relatively new multivariate data analysis that assumes data is non-Gaussian and uses data density information to estimate the transform [21].

Vernat-Rossi *et al.* [22] studied the ability of six tin oxide semi-conductors (FOX 2000, France) to discriminate cured products (dry sausages of various origins or cured hams of different qualities) and also to determine the presence of pathogenic bacterial strains. Using these sensors it was possible to classify 98% of the bacterial strains, 94% of the dry sausages and 87% of cured hams into their respective groups.

Although most applications of metal oxide semi-conductors based sensors in meat have been focused on rapid methods for detecting spoilage by bacterial contamination, some work has also been conducted to determine the presence of off-flavors in meat. In many countries pork production from intact, *i.e.*, non-castrated males, is discouraged because of the unpleasant cooking odor known as “boar taint”. 5a-Androst-16-en-3-one (5a-An) has been identified as the main compound responsible for the urine like-odor associated with boar taint [23], with 3-methylindole (“skatole”) and indole, produced as a result of the metabolism of tryptophan in the intestine, also associated with the taint. There have also been some efforts to identify the main thermally generated volatile compounds that are related to the presence of androstenone [24]. Bourrounet *et al*. [25] used 14 commercially available MOS sensors to classify fat samples from intact male pigs. Samples were first separated in two classes according to the levels of androstenone estimated using ELISA assay, <0.7 μg/g and >1.7 μg/g, respectively. Statistical analysis demonstrated that MOS sensors could classify 84.2% of the samples in two classes.

### 2.2. Fish

Freshness is the most important factor for fish quality. Traditionally, fish quality evaluation has been based on organoleptic tests. This type of testing is subjective even when performed by experienced and well-trained personnel. Gas chromatography has revealed that many volatile compounds are released from degrading fish, some of which can be used as indicators of spoilage. Electronic nose are suitable instruments for measuring fish freshness since a large number of volatile compounds are related to “offness” [26]. Olafsdottir *et al.* [27] employed six MOS sensors (“FishNose”, AlphaMOS-France) to evaluate cold smoked Atlantic salmon and compared the results with sensory analysis and microbial counts. In this research salmon were obtained from smokehouses in Norway, Iceland and Germany and stored in different packing for up to four weeks. Samples were also submitted to chemical analysis including total fat, salt content, water content and chloride content. Partial least square regression modeling, with gas sensors as predictors and sensory attributes as response variables, showed that there is a general correlation between gas sensors and “off-odor” and “sweet/sour” odor attributes. However, the correlation with the chemical parameters was low which meant that they could not be used to calibrate the “FishNose”. With respect to microbial analysis, one sensor type, “PA/2”, showed a very similar pattern to the total viable microbial counts. This work demonstrated that an E-nose could be used for fast quality control and freshness evaluation of smoked salmon products related to microbially produced volatile compounds.

The same E-nose was later used to control processing of smoked salmon in a production plant [28]. It was necessary to correct the sensor reading in this case due to the varying ambient air conditions at the plant. The results showed classification rates ranged between 93% and 95% of fresh samples whereas, for the aged samples, the rate ranged from 81% to 93%. It was not disclosed if these percentages are acceptable for quality control purposes or whether the instrument has been adopted for quality control by the salmon industry.

**Figure 1.**
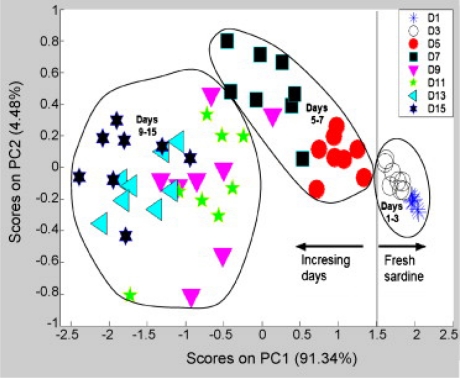
Scores plot of a PCA on sardine data using the six-element gas sensors array. A vertical line separating fresh samples from medium and aged ones and ellipses grouping fresh, medium and aged samples had been added for easy identification (reprinted from [29] with permission from Elsevier, copyright 2007).

Sardines have also been the subject of research with the metal oxide semi-conductor sensors. El Barbri *et al.* [29] developed a simple electronic nose based on commercially available metal oxide sensors in order to monitor the freshness of sardines stored at 4 °C. Principal component analysis (Figure 1) showed that sardine samples could be grouped according to freshness (“fresh”, “medium”, “aged”). This classification was directly related to the number of days that sardines were stored. Other supervised techniques were applied successfully corroborating the usefulness of the sensors to classify samples. The same authors [30] later incorporated a dedicated real-time data acquisition system based on a microcontroller and portable computer in order to miniaturize the device.

### 2.3. Milk and Dairy Products

It is in the area of milk and other dairy products that there has been extensive research in evaluating electronic noses for monitoring the quality of these products. Areas of research have ranged from detecting adulteration/contamination of milk to determining the geographical origins of cheese. A list of reported applications is given below.

#### 2.3.1. Adulteration/ contamination of milk and off-flavors

Liquid milk is an essential nutritional food for infants. Adulteration of milk with water is a matter of serious concern because of the lower nutritional value provided to consumers. The dairy industry employs various quality checks which include the determination of fat and total solids by chemical or physical analyses; estimation of sediment; determination of bacterial count, determination of freezing point, protein, *etc.* [31]. However, most of these measurements are expensive and time consuming since milk samples need to be taken to a laboratory for testing. Yu *et al.* [32] monitored the adulteration of milk using an E-nose (PEN2, Germany) containing ten different metal oxide semi-conductor sensors. Whole milk, reconstituted milk powder and whole milk adulterated with different proportions of water or reconstituted milk powder were followed in storage for 7 days at 20 °C. In this study, the E-nose was able to discriminate skim milk adulterated with different volumes of water and reconstituted milk, and also able to discriminate 100% skim milk samples between 1 and 4 days of storage. However it was not able to discriminate samples between 5 and 7 days of shelf life.

Another area related to milk quality and safety is the detection of contaminants, including aflatoxins, in milk. Benedetti *et al.* [33] studied the feasibility of using a commercial sensor array system, comprising 12 MOS and 12 MOSFET sensors, to detect the presence of aflatoxin M1 (AFM1). The E-nose classification was in complete agreement with aflatoxin M1 content measured by an ELISA procedure. An additional advantage of this approach is that it can be applied to rapid screening for AFM1 contamination in random samples taken at various places through a lot or sub-lot. Samples which are identified as contaminated in the screening process can then be sent for further characterization using quantitative analytical methods, or else rejected to avoid contamination of the entire lot [33]. Factors which are known to the development of oxidized off-flavor in milk products include the contamination of milk with copper, iron, rust and chlorine, or exposure to sunlight as well as excessive incorporation of air. Other off-flavors in milk may be derived from excessive heating which can especially cause certain proteins (such as whey proteins) to burn. Whey proteins are a rich source of sulfide bonds which can form sulfhydryl compounds which can then contribute to off-flavor [34]. Great efforts have been devoted to the optimization of ultra high temperature (UHT) milk processing in order to avoid this effect. One study using an E-nose comprising of MOSFET, MOS and quartz microbalance (QMB) sensors found that it could discriminate as little as 10% boiled milk in pure UHT milk whereas a sensory panel, in contrast, could not discriminate proportions of UHT below 30% [35]. QMB are sensors made of tiny discs, usually quartz, coated with materials such as chromatographic stationary phases that are chemically and thermally stable. When an alternating electrical potential is applied at room temperature, the crystal vibrates at a very stable frequency, defined by its mechanical properties. Upon exposure to a vapor, the coating adsorbs certain molecules which increases the mass of the sensing layer and hence, decreases the resonance frequency of the crystal.

#### 2.3.2. Ageing of milk

**Figure 2.**
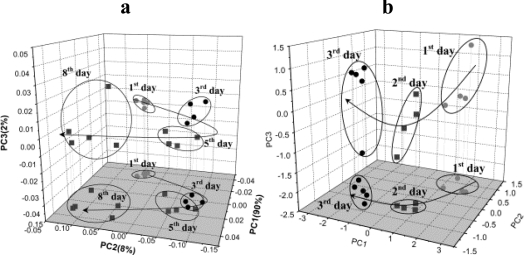
PCA plot based on E-nose measurements of (a) UHT milk and (b) pasteurized milk showing ageing of milk over 8 and 3 days respectively (reprinted from [37] with permission from Elsevier, copyright (2001).

One of most important steps in manufacturing dairy products is the quality control of the starting material. Chemical analysis of flavors in dairy products is often complicated due to the heterogeneous nature of milk. High concentrations of lipids, proteins and carbohydrates in milk can make it difficult to separate flavor-active chemicals based on general properties like polarity or volatility. Furthermore, the headspace of milk typically represents a complex mixture of organic volatiles at varying concentrations and at high relative humidity. Profiling the volatile components in milk and dairy products is usually performed by dynamic or static headspace, “purge and trap” techniques, etc with measurement by gas chromatography-mass spectrometry (GC-MS) [36]. E-noses can also detect milk volatile compounds and are able to monitor the aging of milk [37,38]. An E-nose with five different SnO_2_ thin films, prepared using sol-gel technology, was used to measure the development of rancidity in UHT and pasteurized milk during 8 and 3 days, respectively. The sensors could distinguish between both types of milk as well as determine the degree of rancidity of milks (Figure 2). Labreche *et al.* [39] obtained similar results with an E-nose housing 18 MOS sensors (FOX 4000, France). The E-nose detected significant changes in headspace during milk aging. The authors claimed that there was a high correlation between bacterial counts and the sensor responses.

#### 2.3.3. Ripening of cheese and cheese types

The ripening of Swiss Emmental cheese has been followed with several instruments such as QMB, conductive polymer (CP), mass spectrometer system (MS), MOSFET and a set of MOS [40]. On their own, QMB sensors were not able to discriminate between cheeses ripened between day 1 and 180 days, and the MS was not sensitive enough to detect differences among the samples while the MOSFET was not able to provide good discrimination. However, when the MOSFET was combined with MOS, a good correlation was found with the cheese ripening. Overall, MOS were the most efficient sensors for discriminating among the four stages of ripening. This study also used an unusual system, “trapped flow”, for presenting samples to the E-nose. With trapped flow, the headspace compounds are retained in the sensor chamber for a certain amount of time which allows a better interaction with sensor coatings and consequently stronger responses. However this approach also causes more wear on the sensors. Within less than one year, four of the MOS sensors had to be replaced. The damage was probably caused by the high levels of free fatty acids present in this cheese type and released into the headspace. It was believed that the weak acids react irreversibly with sensor coatings, leading to their accelerated aging. Other workers [41] have used six MOS sensors (FOX 2000, France) to evaluate Swiss type cheeses labeled with different flavors as 0% fat, 33% reduced-fat, sharp, bland and a Norwegian Jarlsburg cheese. The E-nose was able to correctly classify cheeses and it was in agreement with solid phase microextraction-gas chromatography with flame ionization detector (SPME-GC-FID) analysis and sensory analysis.

#### 2.3.4. Lactic acid bacteria

There is a strong interest in exploring the potential of novel *Lactococcus lactis* strains isolated from Nature for the production of aroma in cheeses and other dairy products [42]. A MOS-based electronic nose (FOX 3000, France) with sensory evaluation have been used to evaluate their potential to screen the aroma generation of *Lactococcus lactis* strains as cheese starter cultures [42]. Twenty-three strains of *Lactococcus lactis* were isolated from dairy sources such as boutique raw-milk cheeses, non-dairy sources, and commercial starter cultures (industrial). PCA based on E-nose data revealed four distinctive groups based on aroma profiles that were not correlated with their origin, which also agreed with the results of the sensory analysis.

#### 2.3.5. Off-flavors in cheese

Emmental cheese can develop a “rind taste” off-flavor that can be identified by tasting the cheese at the hoop side (curved side). The components responsible for this off-flavor are often not eliminated during manufacturing, and so may therefore be present in the final product. Cheese loaves must therefore be treated carefully during ripening in order to avoid this problem. The cut pieces are either used fresh or stored in a freezer room which slows down the oxidation process. Attempts to identify the volatile basis of “rind taste” using GC-MS have been unsuccessful [43]. Tainted and untainted Emmental cheeses gave the same profile with the only differences seen being those due to the stage of maturity. A MOS based-E-nose was also totally unable to discriminate contaminated from non contaminated samples. These workers [43] concluded that the rind-related compounds were either not volatiles compounds or were present at levels below the detection threshold of these instruments.

#### 2.3.6. Geographical origin of dairy product

A study on the discrimination of caseinates from different origins was conducted with an E-nose (FOX 4000, France) [44]. PCA and discriminant canonical analysis (DCA) were able to distinguish and classify all samples correctly. The results were validated with measurements of unknown samples obtained from a different supplier and the E-nose showed to be efficient to discriminate the quality between the different suppliers. Similarly, milks from different origins (different brands) that had been pasteurized using similar process were easily distinguished by the same E-nose [45].

### 2.4. Eggs

One of the main concerns of the egg industry is the systematic determination of egg freshness, because some consumers perceive variability in freshness as lack of quality [46]. The modern poultry industry is not satisfied with the traditional system for the handling and processing of eggs, which is based on visual inspection of eggs, mainly because it is time consuming and is not error-free. The industry is therefore interested in evaluating alternative ways that can be used to measure quality parameters more quickly. The main quality parameters of interest are: freshness, weight and shape, state of the eggshell, size of air cell, albumen and yolk quality, the ratio of albumen weight to egg weight (Haugh unit) and eggshell thickness [47]. An alternative strategy for determining the state of freshness of eggs is to detect the organic volatiles emitted by eggs with an E-nose. Methyl sulfide-compounds are good candidates for freshness determination because they are directly related to deterioration and perception of unacceptable odors in whole eggs [48]. Dutta *et al.* [47] used four tin-oxide odor sensors (Figaro Engineering Inc, Japan) to classify eggs non-destructively. Three different freshness categories were identified using PCA which was in good agreement with the three categories of egg freshness determined from the ‘use by date’ of the egg samples. Ninety-five percent classification accuracy was achieved using a radial basis function network.

**Figure 3.**
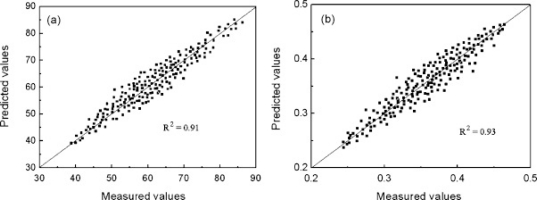
Predicted *versus* E-nose-measured values of egg quality indices from quadratic polynomial step regression models (a) Haugh unit and (b) yolk factor (reprinted from [46] with permission from Elsevier, copyright 2009).

Suman *et al.* [49] compared the performance of an E-nose based on metal oxide semi-conductors with classical chemical and sensory methods for quality control of manufactured egg products. A trained sensory panel evaluated egg samples against the descriptors ‘strong egg odor’, ‘slightly pungent’ and ‘pungent’. Lactic acid and succinic acid in the egg products were determined enzymatically while microbiological analyses included total viable mesophilic bacteria and Enterobacteriaceae counts. The researchers employed a destructive method to evaluate freshness of eggs with E-nose where fresh egg products were spiked and homogenized with different percentages of degraded eggs to obtain different degradation levels. The E-nose demonstrated a high degree of discrimination of samples analyzed during their degradation process compared to chemical and microbiological tests. Wang *et al.* [46] developed prediction models for egg internal quality using an E-nose signal. The E-nose was able to distinguish different storage times under cool (4 °C) and room temperature conditions (21 °C). Moreover, prediction models of the main indicators of internal quality of eggs *i.e.*, Haugh unit and yolk factor (ratio of yolk height and width), using E-nose responses indicated a good prediction performance (Figure 3). Haugh unit had a standard error of prediction (SEP) of 3.74 and a correlation coefficient of 0.91; the yolk factor model had a SEP = 0.02 and correlation coefficient of 0.93 between predicted and measured values respectively.

### 2.5. Grains

Aflatoxins and deoxynivalenol (DON) are highly toxic and carcinogenic secondary metabolites produced by fungi, mainly the genus *Aspergillus* and *Fusarium*. These toxins are found regularly in food commodities, including cereals for human and animal consumption. Warm and/or humid storage conditions for cereals can result in the growth of toxigenic fungi and synthesis of metabolites including dangerous mycotoxins [50]. Because of the highly heterogeneous distribution of mycotoxins in contaminated food supplies, there is a need for cost-effective analytical methods that can process multiple samples from the same lot. Headspace analysis using sensor array appears to be a promising solution. Campagnoli *et al.* [50] employed a commercially available E-nose with ten metal oxide semi-conductor sensors to discriminate between mycotoxin contaminated and un-contaminated cereals. Two of the sensors had partial correlations with the level of aflatoxins in maize. Other two sensors showed high sensitivity towards wheat samples with DON. In both cases 100% correct classification was obtained using linear discriminant analysis (LDA). In another research work, Olsson *et al.* [51] demonstrated that GC-MS system predicted ochratoxin OA concentrations in barley grains with a higher accuracy than ten MOSFET sensor and six SnO_2_ Taguchi sensors, since the GC-MS misclassified only 3 of 37 samples and the electronic nose 7 of 37.

Other applications of E-nose to grains include measuring off-odors indicative of past or ongoing microbial deterioration [52,53]. Borjesson *et al.* [52] tried to classify samples of wheat, barley and oats based on sensory attributes ‘moldy/musty’, ‘acid/sour’, ‘burnt’ and ‘normal’. These workers used ten metal oxide semi-conductor field effect transistor (MOSFET) sensors and four different SnO_2_ semi-conductors. Prediction of the degree of ‘mold/musty’ odor was also included in the study. The E-nose correctly classified 75% of samples according to the four descriptors. Ninety percent of the samples could be correctly assigned using a two-class system ‘good’ and ‘bad’. These values exceed the levels of agreement between two human grain inspectors classifying the same samples. Jonsson *at al.* [53] confirmed these results using the same set of sensors. Sensors could predict the following odor attributes: ‘good’, ‘moldy’, ‘weak musty’ and ‘strong musty’ in oats with high accuracy (100% good prediction). In wheat, correlations of 0.99, 0.84 and 0.88 were found between measured and predicted levels of fungal colony-forming unit (cfu), bacterial cfu and ergosterol in μg g^−1^, respectively.

### 2.6. Fruits

The aroma of fruits and vegetables are either formed during ripening or upon tissue disruption, which occurs after maceration, blending or homogenization. Many volatile compounds are naturally formed by enzymes found in the intact tissue of fruits and vegetables. They originate from secondary metabolites with various biosynthetic pathways. The characteristic aroma of fruits is an important factor in their overall acceptance by the consumer. For many years human senses have been the primary “instrument” that has been used to determine fruit quality. More recently, techniques such as gas chromatography-mass spectrometry (GC-MS) have been used to characterize the volatile profiles of fruits and vegetables. However, it is neither feasible nor practical to use techniques such as GC-MS or sensory panel to assess cultivars or product found at storage stations. Consequently, E-noses have the potential to fill this gap since they are a rapid, transportable and an objective measurement tool for aroma analysis. Gomez *et al.* [54] evaluated the capacity of ten metal oxide semi-conductor sensors (PEN2, Germany) to monitor the change in volatile production during tomato ripening. The E-nose was able to discriminate among different ripeness stages (“unripe”, “half-ripe”, “fully-ripe” and “over-ripe”) with LDA showing 100% correct classification of samples. In the same way, an EOS^835^ E-nose with six thin film MOS sensors was tested for its ability to determine microbial contamination in canned peeled tomatoes [55]. Tomatoes were artificially contaminated with different microbial flora and analyzed by electronic nose, and the E-nose was able to detect contamination at early stages depending on the type of contamination (e.g. *Saccharomyces cerevisiae* and *Escherichia coli*) as well as classify spoiled tomato samples with high fidelity.

Other fruits including blueberries, melons, snake fruit and mandarins have been evaluated by MOS-based electronic noses either to predict the optimal harvest day (OHD) or to monitor shelf life. Blueberry is a highly perishable fruit that must be processed properly and with care otherwise it can develop damage such as cracks, leaks, soft spoilage, which will be apparent to the consumer. Simon *et al.* [56] were able to detect 5% soft and damaged blueberry fruit in packaged containers with just two tin oxide gas sensors and could also distinguish four of five fruit ripeness classes. The sensor responses correlated well with other quality indicators like berry firmness, pH titratable acidity, and color. These workers also detected differences among ten cultivars. In a similar study on snake fruit, three out of 18 MOS-based E-nose (FOX 4000, France) were found able to discriminate among maturity levels [57]. For mandarins however, a sensor array of ten different metal oxide semi-conductor sensors (PEN2, Germany) was not effective in separating mandarins based on different storage times [58]. In contrast, though the same E-nose could detect differences in mandarins picked on different dates and also evaluate mandarin quality attributes such as firmness, soluble solids content and acidity [59]. In a different study with melons and using another E-nose, MOS sensor responses were found to be highly correlated with the sugar content of melon samples harvested at different stages, demonstrating that it could be used to predict the OHD [60,61]. OHD is also of importance in those fruits which are used for further processing, like winegrapes. Berna *et al.* [62] suggested that a MOS based-E-nose (FOX 3000, France) may be a useful practical tool to estimate the time to ripeness of grapes. Preliminary results demonstrated that the E-nose can predict the geographical origins of winegrape samples from five South Australian valleys. LDA of the E-nose responses showed that 87% for both Cabernet Sauvignon and Riesling juice were correctly classified [62]. Likewise, an E-nose based on 12 MOS sensors was successful in distinguishing among Spanish wine grape juice varieties, and between red and white wine grape juices [63,64].

### 2.7. Olive Oils

Virgin olive oils (VOO), and in particular, extra-virgin olive oils (EVOO), are produced using cold pressing techniques. They are sought-after olive oil product because of their aroma, taste, antioxidant, and nutritional properties. The cultivation of olive trees, harvesting of the fruit, and extraction of olive oil are labor intensive and time consuming tasks which add considerably to the overall cost of the oil. Attempts to adulterate VOO with less expensive vegetable oils or lower quality olive oils are thus by no means rare. Not only does this practice defraud consumers, but also constitutes a threat to the reputation and economic development of Mediterranean agricultural communities [65]. Aroma is a fundamental component of the sensory quality of olive oils but sensory panels cannot be used to assess the aroma due to the large number of samples that need to be screened. Gonzales *et al.* [66] used an array of metal oxide semi-conductor gas sensors (FOX 3000, France) to discriminate VOO from non-VOO. The test set comprised 141 commercially available oils of various origins and from different suppliers. Of these samples around 29% were labeled as VOO. Linear discriminant analysis (LDA) applied to the responses of the MOS sensors was able to classify olive oils with 95% of accuracy, indicating that the sensors could determine adulteration of olive oil products.

Other common problems in olive oils are defects caused by chemical taints, mainly volatiles. These may be formed from a number of sources. These include oxidation of unsaturated fatty acids, overripe fruit, molds or bacterial contamination. Trained panels are commonly used to evaluate these defects. Indeed, regulators in the European Union and the International Olive Oil Council (IOOC) [67] have adopted a common standard for assessment of VOOs. The sensory characters of VOO are classified by the IOOC into positive and negative attributes. Negative attributes included: “fusty”, “musty”, “muddy sediment”, “vinegary”, “metallic” and “rancid”. Garcia-Gonzalez *et al.* [68] studied the detection of “vinegary” attribute, produced by acetic bacteria which grow during the storage of olives, using an 18 MOS (FOX 4000, France); six of these sensors were undoped and the remainder doped with metals to shift their selectivity spectrum. The array was trained on a set of Spanish VOOs spiked with different concentrations of acetic acid while the test set comprised Italian VOOs spiked with a standard vinegary VOO supplied by the IOOC. For both varietal VOOs, there was a good linear relationship between levels of acetic acid and the sensor responses (R^2^ = 0.97 and 0.98 for Spanish and Italian VOO, respectively). The same E-nose was evaluated for its capacity to detect other attributes in olive oils like “rancid” and “fusty” [69]. In this case degraded samples were prepared by placing VOO samples under ultraviolet light for up to 12 days or by the addition of different concentrations of a “fusty” standard supplied by IOOC. Correlations of 0.91 for the “fusty” and 0.85 for the “rancid” attribute were found between E-nose sensor responses and sensory evaluations. Using LDA with sensor responses, it was possible to classify defective samples. Samples characterized by the vinegar attribute were clearly distinguished from rancid and fusty samples, a small overlap was observed between fusty and rancid defective samples. The percentage of misclassification was not disclosed.

### 2.8. Alcoholic Drinks

While metal oxide semi-conductors are probably the most widely used of the E-nose sensors, they have still some significant limitations when applied to alcoholic drinks. MOS responses depend logarithmically on the concentration of analyte gases. In the presence of very high concentrations of analyte, such as ethanol in alcoholic beverages, sensors become saturated and mask the responses to other volatile compounds. Consequently, samples tend to be differentiated on the basis of variations in ethanol content rather than the other volatile compounds which are responsible for the aroma [70]. Several strategies have been developed to decrease ethanol content and increase concentration of other volatile compounds before E-nose analysis. These include: the use of a chromatographic column between the headspace sampler and the sensor array to eliminate ethanol [71], and a purge and trap step to eliminate water and ethanol [72]. Other techniques include solid phase micro-extraction (SPME) [73], pervaporation [74,75] or dynamic headspace sampling using resin [76]. Other researchers have successfully dried wine samples on nylon membranes prior to E-nose analysis [77–80]. Beer samples pose an additional problem due to the super-saturation of CO_2_ and foam formation when the samples are heated for headspace sampling. Different approaches have been tested to liberate CO_2_ such as NaCl addition at low temperature [81], nitrogen sparging [82] or simple agitation [83]. Notwithstanding the special challenges presented by alcoholic beverages, E-noses have been used widely to analyze alcoholic beverages, as described below.

#### 2.8.1. Discrimination of wines by denomination of origin and vineyard

**Figure 4.**
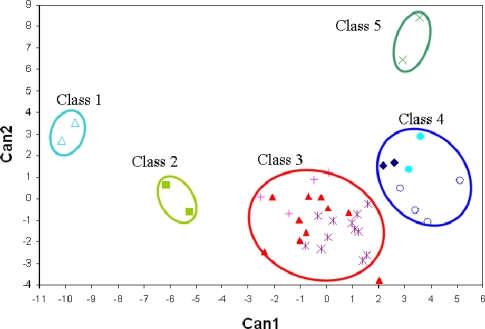
Score plot of the first two canonical variables of the linear discriminate analysis based on the intensity of mass to charge ratio of 34 wines. The symbols represent the six geographical origins of Sauvignon Blanc wines samples. Loire (♦), Marlborough (▴), South Australia (Δ), Tasmania (•), Victoria (+), Western Australia (○) (reprinted from [80] with permission from Elsevier, copyright 2009).

Discrimination of wines is not an easy task due to the complexity and heterogeneity of the headspace. However, classification of wines is an economically important application because of high value of wines from specific geographical regions and also the need to prevent illegal substitution or adulteration. Current methods to evaluate wines include sensory analysis but, because of its high cost, E-noses have been evaluated as an alternative. Penza *et al.* [84] tested three red, three white and three rosé wines from different Italian denominations of origin and vintages using a multisensor array that incorporated four metal oxide (WO_3_) semi-conductor thin film sensors. Static headspace sampling (SHS) was used to sample the volatile compounds above the wine sample. Although SHS, as recognized by the authors [84], is very sensitive to volatile compounds like ethanol and scarcely able to detect trace compounds, it is also a simple, fast and reproducible sampling method that can be used with an automated extraction process. Furthermore it is also low-cost and a solvent free method. Neural network classification of the E-nose data correctly predicted 100% of the white wines, 77.8% of the red wines but only 33.3% of rosé wines. Other researchers [85] employed SHS followed by dynamic headspace to classify Spanish wines according to their geographic origin. Multivariate statistical analysis showed that all the tested wines were different. Buratti *et al.* [86] employed ten metal oxide semi-conductors type chemical sensor (PEN2, Germany) and an enrichment desorption unit to trap and thermally desorb wine samples into the E-nose. The sorbent material was Tenax®-TA polymer and the wine samples came from two northern Italian regions. LDA applied to a larger data set (*i.e.*, chemical analysis, E-nose, Etongue and color measurements) correctly classified 100% of wines into their region. The error rate using only E-nose responses was not disclosed. Berna *et al.* [80] compared E-nose (12 MOS sensors, FOX 3000) SPME measurements after drying wine samples on a nylon membrane with SHS for predicting the regional origins of 34 Sauvignon Blanc wines. GC-MS was also used to analyze all wine samples. LDA applied to GC-MS data showed that there were three distinct clusters or classes of wines with different aroma profiles (Figure 4). After training the E-nose based on GC-MS grouping of wines, the average error of prediction was 6.5% with SPME compared with 24% using SHS.

Di Natale *et al.* [87, 88] employed four MOS sensors to classify wines having the same geographic origin but coming from different vineyards. The E-nose was found superior to the standard chemical analysis routinely executed by the wineries. The standard analysis failed to distinguish classes mainly characterized by the same amount of free SO_2_.

#### 2.8.2. Aging of wines and beers

Aging of wines and beers allows desirable flavors and aromas to develop and off-flavor notes to diminish. Volatile chemicals are an important component of wine and beer flavor and it is desirable to monitor them through aging. Garcia *et al.* [72] analyzed wines of the same variety and geographical origin that differed in age after fermentation (young wines, aged for a year, aged for 18 months and aged for 24 months). They compared two sampling systems, SHS and dynamic headspace with purge and trap, with a 16 MOS sensor array. With SHS sampling 80% of the wines were correctly classified while with purge and trap, a technique more efficient in decreasing moisture and ethanol content in the headspace, the success rate was 95%. More recently, Villanueva *et al.* [89] used 20 MOS sensors combined with SPME to discriminate wines aged for 3 and 6 months in oak barrels. The E-nose clearly distinguished between aging times and was in good agreement with chemical analysis and sensory panel evaluations. McKellar *et al.* [77] attempted to determine the influence of aging time on the development of aroma characteristics of a commercial beer (“Sleeman Cream Ale”) with an E-nose (FOX 3000, France). SHS was the sampling method of choice. The E-nose was able to separate beer samples into distinct groups with those aged up to 12 or 14 days separated from samples aged longer than 14 days. These findings were confirmed by GC-MS. Aging beer for longer than 5 to 7 days appears to have no significant advantage in removing fermentation odors. The authors concluded that reducing the time required in the aging tank would reduce costs to the brewer.

#### 2.8.3. Classification of alcoholic drinks

**Figure 5.**
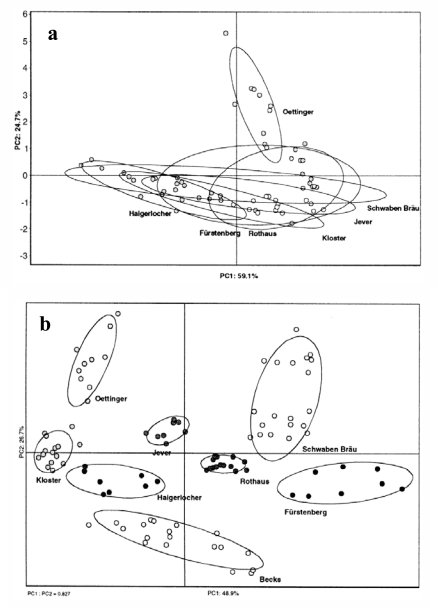
Score plot of eight different beer brands measured with a MOSES II E-nose (a) without pre-separation unit and (b) with pre-separation unit consisted of thermostated gas chromatography column installed between the headspace sampler and the sensor array (reprinted from [71] with permission from Elsevier, copyright 2000).

McKellar *et al.* [78] used a MOS based-E-nose (FOX 3000, France) for the classification of eight fruit wines (blueberry, cherry, raspberry, blackcurrant, elderberry, cranberry, apple and peach) and four grape wines (red, Chardonnay, Riesling and ice wine), and each variety of wine obtained from a minimum of five Ontario wineries. The wine samples were dried onto membrane filters to remove ethanol. Each of the 12 different wine varieties could be separated according to winery. The E-nose appeared to be distinguishing volatile patterns that were characteristic of each winery or the wine-making procedure. However, the E-nose was less able to separate the 12 varieties of wines from each other, in part owing to the variation among wineries. Although the E-nose failed to find dissimilarities between fruit wines, the same type of E-nose (FOX 2000, France) seems to be more efficient to discriminate between other alcoholic beverages like tequila, whisky, vodka, wine and beer as found by Ragazzo-Sanchez *et al.* [90]. The authors used gas chromatogram (GC) to remove ethanol and dehydrate headspace beverages with the remaining volatile compounds collected by reverting the gas using column ‘back-flush’ and introduced in the injector port of the E-nose. PCA analysis showed that most beverages were correctly discriminated, except for some confusion between tequilas and whiskies. Interestingly, the order of the classification along the PC axis was not related to the initial ethanol content. Aishima *et al.* [73] succeeded in classifying eight liquors (shochu, white and red wines, beer, sake, whiskies and cognac) with six semi-conductor gas sensors. In this case volatiles were pre-concentrated in a Tenax®-TA trap to remove ethanol. Others [71], using chromatographic preseparation, have characterized the ‘flavor peaks’ of different beer brands using a modular sensor array set up consisting of eight quartz microbalance (QMB) and eight SnO_2_ based MOS sensors (MOSES II, Germany). Without the removal of the ethanol content (similar in each sample), it was impossible to discriminate beer brands (Figure 5a). However after pre-separation, beer brands could be perfectly distinguished (Figure 5b). Similar results were found by Ragazzo-Sanchez *et al.* [91] using 18 MOS E-nose (FOX 4000, France) and sample agitation to reduce CO_2_ from headspace. The E-nose successfully discriminated three classes of beers even though the beer samples were spiked with off-flavors.

#### 2.8.4. Detection of aromatic compounds and off-favors in wines

A wine fault or defect is an unpleasant characteristic of a wine often resulting from failures in winemaking or storage conditions. Normally, many of the compounds that cause wine faults are present in wine at concentrations which do not adversely affect it. However when these concentrations exceed the sensory threshold, the resulting aroma can overwhelm or obscure the desirable flavors and aromas of the wine. The quality of the wine is reduced, making it less appealing and sometimes undrinkable. Some of the most common off-flavor metabolites are produced by *Brettanomyces* yeasts: 4-ethylphenol (4EP) and 4-ethylguaiacol (4EG). Typically these taints are described as “barnyard”, “sweaty saddle” and “band-aid” when present in red wine at concentrations of several hundred μg L^−1^ or more. Using nylon membranes to remove ethanol, Berna *et al.* [79] established that the detection limits for a MOS-E-nose (FOX 3000, France) were 44 μg L^−1^ for 4EP and 91 μg L^−1^ for 4EG (Table 2); these values are significantly lower than the reported human sensory thresholds of 605 and 110 μg L^−1^ for 4EP and 4EG, respectively [92]. Partial least squares (PLS) regression of electronic nose signals against the known levels of 4EP and 4EG in 46 Australian red wines showed that the MOS-E-nose was unable to identify *Brettanomyces* spoilage reliably due to the gas sensors responding to inter-sample variation in volatile compounds in the samples rather than the ethylphenol content. It was concluded that, following ethanol removal, existing metal oxide sensors are sufficiently sensitive to detect Brett taints in wine but lack the selectivity needed to perform this task when the aroma volatiles background varies. This lack of selectivity was confirmed later by Ragazzo *et al.* [91] who attempted to predict the presence of off-flavors like, ethyl acetate (“oxide” attribute), 2,4,6-trichloroanisole (“musty/cork” attribute), 4EP (“barnyard” notes) and hexanol (“herbaceous” attribute), in wines using 18 MOS sensors (FOX 4000, France). If the origin of the wine was known the prediction error of contaminated wines was 11% however, if the wine was of unknown origin the E-nose failed to detect the off-flavor. In another work, a 16 MOS based E-nose could discriminate different aromas spiked in a base white wine with an accuracy of 97.2% [93]. Others found that, when comparing sensory human panel thresholds with chemical MOS sensors to detect wine-related aroma compounds, the perception level of the human nose was superior to the E-nose [94,95], but the E-nose had a better recognition threshold in recognizing a specific odor [94].

### 2.9. Non-Alcoholic Beverages

Tea and coffee are the most popular beverages which have been the subject to research using electronic noses, particularly in the evaluation of the quality grades of these products. Because of the complexity of the organic compounds present in both raw materials, E-noses are suitable for continuous real time monitoring of odor. Tea grade is traditionally classified by a trained human panel. In total, 24 non-overlapping flavor terms have been identified out of 40 generally used flavor notes [96]. Dutta *et al.* [97] assessed an E-nose for its suitability in monitoring the quality of five Assam tea samples manufactured under different processing conditions in India. Tea sample variations were based on drying of the product, fermentation and the final oven fired process. Four tin oxide sensors (Figaro Eng. Inc, Japan) were used to evaluate headspace of liquid tea samples. MOS sensors were able to discriminate five different categories of tea, indicating that the instrument was able to discriminate between flavors of teas manufactured under different processing conditions. A probabilistic neural network used with the E-nose responses provided 100% accuracy in classification of the tea samples. Yu *et al.* [98] studied the applicability of an electronic nose for assessing the same category of tea with different quality grades. Tea samples had five grades (different prices) picked from the same area and prepared as tea leaf or tea beverage or tea remains. The E-nose housed ten different metal oxide semi-conductor sensors (PEN2, Airsense Analytics) and LDA of the sensor responses showed correct classification ratios of 93.3 % and 100% for tea leaf and tea beverage samples respectively. For tea remains the classification results were not disclosed but appeared to be not as good as the other two samples.

Like tea, coffee quality is assessed by coffee tasters, largely on the basis of its aroma and flavor. The highest quality beans command a considerable premium when the product is sold. Coffee volatiles are numerous and varied in their aroma quality, potency and concentration. Most of the volatiles are derived from non-volatile components of the raw bean, and formed during roasting to generate a complex aroma mixture. Green coffee beans are generally regarded as having no agreeable aroma or flavor but do possess a large number of volatiles, most of which increase in concentration during coffee roasting even though there is a minority which tend to degrade [99]. Aishima *et al.* [100] focused on the ability of six MOS sensors to discriminate coffee beans and instant coffee. They found, after cluster analysis applied to the normalized responses, a clear separation between coffee beans and instant coffees. Stepwise LDA applied to the data matrix showed that a single sensor (TGS812) could effectively discriminate coffee aromas. On its own that sensor correctly classified 78% of samples. When four sensors were included in the model the classification could perfectly discriminate among two ground coffees and two instant coffees. Correlation coefficients between sensor responses ranged between 0.90 and 0.99 explaining the cross sensitivity of the sensors. Gardner *et al.* [99] employed an array of 12 tin oxide sensors to evaluate three commercial coffees (covering two different blends and two roasted varieties) as well as one coffee sample which was subjected to a range of six roasting times. For the three commercial coffees, a success rate of 89.9% was achieved when using the entire data set but this value decreased to 81.1% when half of the data set was used for cross-validation. The success in predicting roasting time was 88.1%. It seemed that tin oxide gas sensors were suitable for discriminating between both the blend and roasting level of coffee, confirming the potential of E-noses for on-line quantitative process control in the coffee industry.

Other application of E-noses to coffees include the study of Pardo *et al.* [101] who evaluated a system consisting of four SnO_2_ thin film sensors, of which two were pure SnO_2_, another doped with gold and the last one doped with platinum, to distinguish between commercial coffee blends. Twelve types of coffees were evaluated in the form of espresso extract and each were sampled at three points in preparation: as beans, ground powder and liquid. Only two sensors were needed to correctly classify 100% of the bean samples. In the case of ground coffee, a supervised drift compensation algorithm was developed and 87.5% correct classification was achieved. On the other hand, liquid coffee samples were not successfully classified; which the authors attribute to the difficulty in assuring reproducible sampling conditions. In later work Pardo *et al.* [102] used the same system with an extra SnO_2_ sensor doped with palladium in order to evaluate two groups of coffees. The first group comprised six single coffee varieties and an Italian certified espresso blend while the second group of coffees consisted of seven blends. The E-nose results were then compared with sensory analysis of the final product; *i.e.*, cups of espresso, with the panel judging ten quantitative descriptors and four qualitative descriptors. Using PCA and multilayer perceptrons, 82% of the samples were correctly classified for the first group of coffee while for the second group, the classification rate was 87%. Moreover, MOS sensor responses were used to predict single quantitative descriptors and the best predicted descriptors were “global negative odor” and “global positive odor”, with correlation coefficients of 0.90 and 0.89, respectively.

Falasconi *et al.* [103] used a novel Electronic Olfactory System EOS^835^ (Sacmi Imola, Italy), housing six thin-film semi-conductor MOS, to monitor the ripening or seasoning process of commercial coffee blends, made from 12 different types of monocultivar *Arabica* coffee. Although the authors focused on the investigation of sampling conditions and feature selection for improving classification performance, the results showed that EOS^835^ was suitable to monitor coffee blends during the seasoning process. Only two sensors performed adequately for this application.

### 2.10. Other Food

Electronic noses have also been evaluated for their ability to measure shelf life of products like nuts and fresh cut vegetables [104,105]. Because they have high fat content and therefore are susceptible to rancidity arising from lipid oxidation nuts are a good model product for shelf life studies [106]. Storage variables such as light and temperature can influence the development of off-flavors. Hexanal is the main aldehyde formed during oxidation of unsaturated fats and therefore is a good representative marker of oxidative rancidity [10]. An electronic nose with ten metal oxide semi-conductor sensors (PEN2, Germany) was compared with GC-MS for the estimation of hexanal formed in hazelnuts during storage under different conditions (room temperature, 40 °C, ultraviolet light, with and without oxygen scavenger) [105]. The results obtained with the two instruments correlated well. The E-nose discriminated nut samples stored under UV light without oxygen scavenger from the rest of samples. Differences were not observed between samples stored at 40 °C and at room temperature. Metal oxide semi-conductors based E-noses have also been used to shelf life studies of fresh cut vegetables [104]. Minimally processed vegetables (MPV) are fresh raw vegetables that are sold ready-to-use. They are usually peeled, washed, dried and are sometimes cut and packaged in either sealed pouches or wrapped trays. The degradation of MPV is a complex process which includes microbial spoilage and a number of physico-chemical and bio-chemical modifications that mainly affect the sensory properties of the product. Spoilage may be detected through the analysis of off-odors produced by bacteria. Riva *et al.* [104] used an E-nose equipped with ten MOSFET and five MOS sensors to evaluate shelf life of ready-to-use fresh cut chicory (*Cichorium intybus*) and carrots (*Daucus carota*). PCA of the data demonstrated that E-nose responses correlated well with classical evaluation of vegetable spoilage, *i.e.*, microbial population and color index. E-nose is therefore a suitable tool for monitoring the storage of these products.

Discrimination of bacterial species is also of interest to the food industry although E-noses have not been extensively investigated in this application. Rossi *et al.* [107] looked at the ability of the E-nose to discriminate six sub-species of *Staphylococcus* and one of *Micrococcus*. These microbes are of interest since they have been reported to be present in fermented sausages [108]. Factorial discriminant analysis of the signals revealed that it was possible to classify 100% of bacterial species into their respective groups. A cross-validation of the discriminant axes classified 90.5% of the bacterial strains correctly.

## 3. Conclusions

Potential applications in odour assessment by electronic noses in the food area are numerous; they have been used for quality control, monitoring process, aging, determination of geographical origin, adulteration, contamination and spoilage. In most cases classification of samples was above 85%, but, before these specific applications can become a reality, *i.e.*, these laboratory-based assays are moved into the industry, a number of challenges still need to be met; these are to properly assess various characteristics of electronic nose performance, including drift, humidity influence, redundancy of sensors, selectivity and signal to noise ratio. Although new sensor materials and designs, and correction algorithms that can be applied for each sensor, are being reported, the major limitation of currently available MOS sensors remains their independence and selectivity. Sensors with poor selectivity affect adversely the discriminating power of the array. Additionally, with the technology developed so far, it is unrealistic to envisage a universal electronic nose that is able to cope with every odour type as specific data processing and, sometimes instrumentation, must be designed for each application.

## Figures and Tables

**Table 1.** Application of MOS based E-noses in the food industry, sensors used and performance.

**Commodity**	**Test**	**No. of MOS sensors**	**Material**	**Commercial (C) or experimental (E) E-nose**	**Ref.**
Meat	Freshness and type of meat	4*	SnO_2_	C	[19]
	Microbiological contamination	7	SnO_2_ thick film	E	[20]
	Origin of meats	6	SnO_2_	C	[22]
	Taints	5	SnO_2_	E	[25]
Fish	Freshness	6	SnO_2,_ CTO, WO_3_	C	[27]
		6	SnO_2,_ CTO, WO_3_	C	[28]
		6	SnO_2_	E	[29]
Milk and dairy products	Adulteration/ Contamination	10	SnO_2_	C	[32]
		12*	SnO_2_	C**	[33]
	Off-flavor	n.d.	n.d.	E	[35]
		12*	SnO_2_	C**	[43]
Milk and dairy products	Aging/ripening	5	SnO_2_ thin film doped with Pd, Pt, Os, Ni	E	[37,38]
		18	SnO_2,_ CTO, WO_3_	C	[39]
		5	n.d.	C**	[40]
	Cheese type	6	SnO_2,_ CTO, WO_3_	C	[41]
	Bacterial strains	12	SnO_2,_ CTO, WO_3_	C	[42]
	Origin of caseinates	18	SnO_2,_ CTO, WO_3_	C	[44]
	Origin of milk	18	SnO_2,_ CTO, WO_3_	C	[45]
Eggs	Freshness	8	n.d.	E	[46]
		4	SnO_2_	E	[47]
		12	SnO_2_ thick film	C	[49]
Grains	Contamination by toxin	6*	SnO_2_	C*	[51]
		10	SnO_2_	C	[50]
	Off-flavors	4*	SnO_2_	E	[52]
		4*	SnO_2_	E	[53]
Fruit	Ripening changes	10	SnO_2_	C	[54]
		2	SnO_2_	E	[56]
		10	SnO_2_	C	[59]
		5	SnO_2_	E	[60,61]
		12	SnO_2,_ CTO, WO_3_	C	[62]
	Varieties	16	SnO_2_ thin film doped with Cr, Pt	E	[63,64]
	Microbial contamination	6	n.d.	C	[55]
	Shelf life	18	SnO_2,_ CTO, WO_3_	C	[57]
		10	SnO_2_	C	[58]
Olive oil	Authenticity	6	SnO_2,_ CTO, WO_3_	C	[66]
	Taints	18	SnO_2,_ CTO, WO_3_	C	[68,69]
Alcoholic beverages	Denomination of origin and vineyard discrimination	4	WO_3_	E	[84]
		16	SnO_2_ thin film doped with Cr, In	E	[85]
		10	SnO_2_	C	[86]
		4	SnO_2_ thin film doped with Pt, Pd	E	[87,88]
	Aging of wines and beers	16	SnO_2_ thin film doped with Cr, In	E	[72]
		20	SnO_2_	E	[89]
		12	SnO_2,_ CTO, WO_3_	C	[77]
Alcoholic beverages	Classification of drinks	12	SnO_2,_ CTO, WO_3_	C	[78]
		6	SnO_2,_ CTO, WO_3_	C	[90]
		6	SnO_2_	E	[73]
		6*	SnO_2_	C	[71]
		18	SnO_2,_ CTO, WO_3_	C	[91]
	Off-flavors and aromatic compounds	12	SnO_2,_ CTO, WO_3_	C	[79]
		18	SnO_2,_ CTO, WO_3_	C	[91]
		16	SnO_2_	E	[93]
		16	SnO_2_ thin film	E	[95]
		16	SnO_2_	E	[94]
Non alcoholic beverages	Grading	4	SnO_2_	E	[97]
		10	SnO_2_	C	[98]
	Quality/process control	6	SnO_2_	E	[100]
		12	SnO_2_	E	[99]
		4	SnO_2_ thin film doped with Au, Pt	E	[101]
		5	SnO_2_ thin film doped with Au, Pt, Pd	E	[102]
		6	WO_3,_ SnO_2_	C	[103]
Other food	Shelf life of nuts	10	SnO_2_	C	[105]
	Shelf life of vegetables	5*	n.d.	E	[104]
	Bacterial species classification	6	SnO_2,_ CTO, WO_3_	C	[107]

* Modular sensor consisting of MOS and quartz microbalance (QMB) or metal oxide semi-conductor field-effect transistor (MOSFET)

** The instrument was commercially available at the time of research publication

n.d. Not disclosed

**Table 2.** Equations of the Linear Regression for the Different Compounds with Correlation Coefficient r^2^ and detection limit of the linear range (reprinted with permission from [79]; copyright 2008 American Chemical Society).

**Compound**	**E-nose sensor type**	**Linear regression: a×conc. + b**			**Detection limit (μg L^−1^)**
		**a**	**b**	**r^2^**	

4-Ethylphenol	SY/G	6.80E-3	−1.02	0.99	101.2
	SY/Gh	6.05E-3	−1.13	0.99	138.4
	SY/gCT	6.08E-3	−1.34	0.99	43.8

4-Ethylguaiacol	SY/Gh	6.75E-4	−0.59	0.81	93.5
	SY/gCT	6.60E-4	−0.37	0.78	91.1
